# Maternal knowledge, education, and attitudes as determinants in preventing stunting: A case study from a rural area in South Sumatra

**DOI:** 10.51866/oa.817

**Published:** 2026-04-28

**Authors:** Nur Alam Fajar, Nura Malahayati, Muhammad Cholil Munadi, Iche Andriyani Liberty, Esti Sri Ananingsih

**Affiliations:** 1 Faculty of Public Health, Sriwijaya University, Indonesia.; 2 Food Biochemistry and nutrition, Faculty of Agriculture, Sriwijaya University, Indonesia.; 3 Department of Public Health and Community Medicine, Sriwijaya University, Indonesia.; 4 Politeknik Kesehatan Kemenkes Palembang, Indonesia.

**Keywords:** Stunting, Maternal knowledge, Child nutrition disorders, Health education

## Abstract

**Introduction:**

Stunting represents a critical global health challenge, particularly in low- and middle-income countries, due to its impact on child development and long-term economic productivity. Despite national efforts in Indonesia, stunting remains prevalent and necessitates localised studies to address this issue. This study aimed to examine factors associated with stunting in rural South Sumatra, Indonesia.

**Methods:**

A cross-sectional study involving 348 respondents was conducted across 11 villages in Musi Rawas Regency, South Sumatra. Data were analysed using logistic regression to identify the dominant risk factors.

**Results:**

Maternal knowledge during pregnancy was significantly associated with stunting prevention, with mothers having lower knowledge showing higher odds of poor preventive practices (OR=1.663; 95% CI=1.149–1.857; P=0.007). Postnatal maternal knowledge also remained a significant predictor (OR=1.479; 95% CI=1.036–2.112; P=0.031). In contrast, maternal attitudes during pregnancy (P=0.356) and after childbirth (P=0.109) were not significantly associated and were not retained as predictors in the final model.

**Conclusion:**

Maternal knowledge, education, and attitudes are pivotal in preventing stunting. Targeted interventions aimed at improving maternal education, awareness and attitudes are essential to mitigating stunting in rural South Sumatra.

## Introduction

Stunting, a condition marked by chronic undernutrition that impairs growth and development, remains one of the most pressing global health challenges. The World Health Organization (WHO) estimated that in 2020, around 22% (149 million) of children under 5 years of age worldwide were affected, with the highest prevalence noted in low- and middle-income countries.^1^ Stunting impairs physical growth and has profound long-term consequences on cognitive development, school performance, and economic productivity in adulthood.^2^

A complex interplay of socioeconomic and environmental factors influences stunting in resource- limited settings. Restricted access to diverse and nutritious food options is critical, particularly in rural areas where food insecurity is widespread.^3^ Lower household income is strongly associated with higher stunting rates, as families with constrained means struggle to provide adequate diets.^4^ Mothers with limited educational attainment are more likely to have children experiencing stunting, as schooling influences health literacy and access to services.^5,6^ Larger family sizes can strain household resources, leading to nutrition for children. Female-headed households also show higher stunting rates,^3^ while poor water, sanitation, and hygiene conditions contribute to frequent infections that exacerbate malnutrition.^5^

In Indonesia, stunting rates remain alarmingly high despite national initiatives to reduce its occurrence. Data from the Ministry of Health that 27.7% of children under 5 years old were stunted in 2019, a figure surpassing the global average.^7^ Although strategies such as the National Strategy to Accelerate Stunting Prevention 2018–2024 have sought to address the issue, implementation varies considerably across regions.

A significant body of research has identified well-established risk factors, including maternal education, sanitation and dietary diversity.^2^ Evidence also highlights the role of inadequate maternal nutrition, low educational attainment, and insufficient healthcare access. These factors underscore the need for targeted interventions that address the underlying determinants at the community level.^2,8^ Although numerous studies have explored stunting in Indonesia, gaps remain concerning the socioeconomic, environmental, and maternal influences in rural contexts such as Musi Rawas Regency.

Located in the western part of South Sumatra Province, Indonesia, Musi Rawas is predominantly rural and reliant on agriculture. Covering an extensive area, the regency is home to diverse communities, as highlighted in this study. The population is primarily engaged in farming activities, with rubber and palm oil plantations serving as the backbone of the local economy. Despite its agricultural wealth, Musi Rawas faces persistent socioeconomic challenges, including widespread poverty and limited educational opportunities, particularly for women. These geographical and demographic factors, combined with dispersed populations and remote villages, present unique challenges for public health interventions, making the region a crucial focal point for addressing stunting and maternal health disparities.

Identifying these determinants is important for designing effective public health interventions and policies that can mitigate the long-term consequences of stunting.^9^ This study sought to investigate the key risk factors associated with stunting among children under 5 years old in a rural area of South Sumatra. By understanding these influences, the study is expected to provide data-driven recommendations that can inform local health policies and contribute to global efforts to reduce stunting in rural populations.

## Methods

This study adopted a cross-sectional design and was undertaken in Musi Rawas Regency, South Sumatra Province, Indonesia, covering 11 villages: Air Beliti, Bamasco, Banpres, Dharma Sakti, Jaya Bhakti, Jaya Tunggal, Leban Jaya, Lubuk Rumbai, Petunang, Remayu, and Sukamulya. The research was conducted over a 5-month period, encompassing proposal development, instrument design, ethical approval, data collection, data processing, statistical analysis, and report writing.

The study population was drawn from the working area of the Air Beliti Community Health Centre. The sample size was determined using Lemeshow’s formula, yielding 348 respondents.

Pregnant women and mothers with children aged 0 - 2 years residing in the stunting locus area of Tuah Negeri Subdistrict, Musi Rawas Regency, were included in study. The exclusion criteria in this study included mothers who refused to participate and those who did not respond to the explanation provided regarding the research procedures; therefore, they were not included as study participants.

The dependent variable in this study was stunting prevention behavior during pregnancy and after childbirth, defined as the actions taken by mothers to prevent stunting in children during the prenatal and postnatal periods. The variable was measured using a structured questionnaire with a scoring system and was categorized into two groups: not preventing (score < cut-off) and preventing (score ≥ cut-off).

Anthropometric measurements were performed by trained health workers who had completed relevant training, following standardised procedures based on the WHO Child Growth Standards.

The independent variables consisted of father’s occupation, mother’s occupation, family income, maternal education, maternal knowledge, and maternal attitude. Father’s occupation was defined as the primary source of income or productive activity to meet family needs, categorised as either employed or unemployed. Mother’s occupation referred to income-generating or productive activity undertaken to meet the needs of the family, likewise classified as employed or unemployed.

Family income represented the total income earned by the family in a certain period, usually per month, which included income from father’s and mother’s work, and other sources where applicable. It was divided into low and high: low if below the minimum wage standard of the study area, and high if above this threshold. Maternal education denoted the highest level of formal schooling completed, which described access to and achievement in education, with potential implications for childcare and parenting capacity. It was grouped into high (if mothers completed high school or above) and low (if mothers completed junior high school or below).

Maternal knowledge referred to mother’s understanding of stunting prevention during pregnancy, including nutrition, maternal health, and factors influencing foetal growth. It was assessed using a structured questionnaire containing several questions related to nutritional needs during pregnancy, the importance of regular antenatal check-ups, and risk factors that may lead to stunting in children, administered through direct interviews with mothers.

Knowledge scores were categorised into low (<5;0) and high (>50), based on the average score. A low score indicated limited awareness, such as minimal comprehension of nutrition, antenatal care, or stunting risks, whereas a high score reflected broader understanding, including the importance of nutritious foods, routine health checks, and avoidance of behaviours that may hinder foetal growth.

Mother’s attitudes included perceptions, beliefs, and inclinations towards the significance of stunting prevention during pregnancy, which may affect preventive actions. Attitudes were measured using a questionnaire containing items on perceptions and beliefs about stunting prevention. They were categorised as unsupportive, where mothers did not prioritise or believe in preventive measures (e.g. considering additional nutrition or antenatal check-ups unnecessary or perceiving stunting risk as insignificant), and supportive, where mothers held positive beliefs and were willing to commit to preventive actions (e.g. ensuring proper nutrition, attending regular health checks, and avoiding activities that increase foetal risk).

Assessment of maternal knowledge and attitudes towards stunting prevention was based on mothers’ experiences during pregnancy and childbirth, which may introduce information bias. Validation through other family members was used to improve accuracy.

A univariate analysis was performed to describe family characteristics, while a multivariate analysis using logistic regression identified the dominant risk factors for stunting, with significance levels of P<0;.05 and a confidence interval (CI) of 95%. The modeling approach used in this study involved stepwise multivariate analysis conducted across three stages (Model 1, Model 2, and Model 3). Model 1 represents the analysis of the independent variables in relation to stunting during pregnancy. Model 2 represents the analysis of the independent variables in relation to stunting after childbirth. Model 3 represents the final model, which is the combined results of the analyses from Model 1 (during pregnancy) and Model 2 (after birth). The independent variables associated with stunting prevention included maternal education, maternal attitudes, and maternal knowledge. Data analysis was conducted using IBM SPSS Statistics version 26 (IBM Corp., Armonk, NY, USA).

## Results

Table 1 shows that 25.9% of the children were identified as stunted. The majority of the fathers (86.5%) were engaged in informal employment, while 74.1% of the mothers were not employed. Household income was low in 62.6% of the families. Regarding maternal education, 37.9% had completed senior high school. During pregnancy, 62.6% of the mothers demonstrated limited knowledge of stunting prevention; 53.2% expressed unsupportive attitudes; and 67% did not take actions to prevent stunting. After childbirth, the mothers’ knowledge level was high (54%), but a considerable proportion (53.4%) continued to exhibit unsupportive attitudes.

**Table 1:** Characteristics of the respondents.

Variable	n	%
Stunting Prevention (during pregnancy)		
No preventive practice	233	67
Preventing	115	33
Stunting Prevention (after pregnancy)		
No preventive	189	54.3
Preventing	159	45.7
Father’s occupational status		
Unemployed	25	7.2
Informal	301	86.5
Formal	22	6.3
Mother’s occupational status		
Unemployed	258	74.1
Informal	71	20.4
Formal	19	5.5
Family income		
Low	218	62.6
High	130	37.4
Maternal educational level		
Primary school	84	24.1
Junior high school	96	27.6
Senior high school	132	37.9
Diploma	29	8.3
Undergraduate degree	2	0.6
Master’s degree	2	0.6
Doctorate	3	0.9
Maternal knowledge level (during pregnancy)		
Low	218	62.6
High	130	37.4
Maternal knowledge level (after birth)		
Low	160	46.0
High	188	54.0
Maternal attitude (during pregnancy)		
Unsupportive	185	53.2
Supportive	163	46.8
Maternal attitude (after birth)		
Unsupportive	186	53.4
Supportive	162	46.6

Table 2 shows the model of factors associated with stunting prevention. In Model 1 (during pregnancy), the findings indicated that low maternal knowledge level and low maternal educational level were associated with stunting. In Model 2 (after birth), the findings indicated that low maternal knowledge level, low maternal educational level, and unsupportive attitude were associated with stunting.

**Table 2:** Multivariate model of the risk factors for stunting prevention.

	Model 1	Model 2	Model 3
Variable	Odds Ratio (95% confidence interval); P-Value	Odds Ratio (95% confidence interval); P-Value	Odds Ratio (95% confidence interval); P-Value
Mother’s educational level			
Low	2.203 (1.377 - 3.535); 0.001	1.606 (1.023 - 2.523); 0.040	1.583 (1.018 - 2.460); 0.041
High	Ref	Ref	Ref
Mother’s occupational status			
Unemployed	0.878 (0.580 - 1.328); 0.537	1.049 (0.708 - 1.556); 0.810	
Employed	Ref	Ref	
Family income			
Low	1.090 (0.669 - 1.776); 0.730	0.880 (0.552 - 1.404); 0.593	
High	Ref	Ref	
Father’s occupational status			
Unemployed	0.793 (0.412 - 1.525); 0.487	0.801 (0.435 - 1.476); 0.477	
Employed	Ref	Ref	
Maternal attitude			
Unsupportive	1.287 (0.802 - 2.065); 0.292	1.616 (1.003 - 2.603); 0.048	1.614 (1.006 - 2.589); 0.047
Supportive	Ref	Ref	Ref
Maternal knowledge level			
Low	2.038 (1.270 - 3.273); 0.003	2.104 (1.306 - 3.389); 0.002	2.080 (1.294 - 3.343); 0.003
High	Ref	Ref	Ref

Model 3 revealed that the mothers with lower educational level, unsupportive attitudes, and limited knowledge were significantly less likely to adopt preventive behaviours. Specifically, limited schooling, negative attitudes, and inadequate knowledge increased the risk of nonpreventive practices by 1.583, 1.614, and 2.080 times, respectively.

Multivariate logistic regression was use to determine the factors linked to stunting preventive practices. Maternal knowledge and attitudes during pregnancy and after childbirth were incorporated into the final model. Table 3 summarises the findings.

**Table 3:** Final model of the risk factors for stunting prevention.

Variable	P-value	Model 1 OR (95% CI)
Maternal knowledge level (during pregnancy)		
Low	0.007	1.663 (1.149 - 2.406)
High		Ref
Maternal attitude (during pregnancy)		
Unsupportive	0.356	1.191 (0.822 - 1.724)
Supportive		Ref
Maternal knowledge level (after birth)		
Low	0.031	1.479 (1.036 - 2.112)
High		Ref
Maternal attitude (after birth)		
Unsupportive	0.109	1.321 (0.940 - 1.857)
Supportive		Ref

### Maternal knowledge during pregnancy

Prenatal maternal knowledge had a strong association with stunting prevention. The mothers with low knowledge levels had 1.66 times higher odds of performing poorly at preventive practices than those possessing greater knowledge (OR=1.663; 95% CI=1.1492 - 1.857; P=0.007). This suggests that knowledge gained during pregnancy is of great importance in the formation of preventive behaviours.

### Maternal attitudes during pregnancy

Attitudes in pregnancy did not have a substantial connection with stunting prevention. Although the odds of having inadequate preventive practices were marginally higher in the case of unsupportive attitudes (OR=1.191; 95% CI=0.822 - 1.724), the relationship was not found to be statistically significant (P=0.356). Therefore, prenatal maternal attitudes were not predictive variables in the final model.

### Maternal knowledge after childbirth

Postnatal knowledge also played a major role in the prevention of stunting. The mothers with limited understanding had 1.479 times greater odds of inadequate practices than those with higher levels of knowledge (OR=1.479; 95% CI=1.036 - 2.112; P=0.031). This finding implies that maternal knowledge is influential in shaping preventive behaviours beyond pregnancy.

### Maternal attitudes after childbirth

There was no significant relationship between maternal attitudes after childbirth and stunting prevention. While unsupportive attitudes were correlated with higher odds (OR=1.321; 95% CI=0.940 - 1.857), the relationship was not statistically significant (P=0.109). Thus, postnatal attitudes were not identified as primary predictors in the final model.

## Discussion

This study explored the variables related to stunting among children below the age of 5 years in rural South Sumatra, Indonesia, where the prevalence was recorded at 25.9%. This figure exceeds the national prevalence reported in the 2018 Indonesian Basic Health Research (Riskesdas), which stood at 30.8%,^10^ slightly lower than the 2022 rate of 24.8%.^11^ At the provincial level, South Sumatra showed the highest prevalence at 24.7%, indicating that the burden in the study area is consistent with the rest of the regions. These comparisons imply that stunting remains a persistent public health challenge in rural settings, despite improvements observed at the national level.^12^

In the current study, there was a strong link between stunting and a low maternal educational level.^13^ This is consistent with other report in Indonesia, which constantly show that less educated mothers are not aware of nutritional needs and growth control.^14,15^ National Riskesdas records also show that children of mothers with only primary education or below are at a greater risk than those whose mothers attained secondary or higher education.^16,17^ Evidence from Magelang Regency further supports this:, Low maternal educational levels increased the risk of stunting by 1.6 times, stressing the continuing role of educational gaps on child growth.^18,19^

The study also demonstrated that limited maternal knowledge was related to stunting. This is consistent with evidence from various regions of Indonesia.^20^ Research in Indonesia continues to highlight maternal knowledge as a strong determinant of stunting, including findings that poor maternal health literacy increases the odds of stunting.^21,22^ These findings reinforce the importance of maternal understanding of complementary feeding, hygiene, and growth monitoring in preventing stunting.^23^

Another major cause of stunting identified in the present study was maternal attitudes.^24^ The mothers who had unsupportive attitudes towards stunting prevention were 1.6 times more likely to have stunted children.^25^ Regional studies confirm this, showing that negative nutritional attitudes among mothers strongly influence child nutritional outcomes, and that supportive parental attitudes significantly improve stunting prevention behaviours.^26^ The relationship between attitudes and stunting is shaped by cultural norms, decision-making power in the family, and societal beliefs, which play a dominant role in rural Indonesian communities.^26,27^

This study’s results reflect the general situation in Indonesia, where maternal multi dimensional factors continue to contribute to stunting.^28^ According to the 2024 SSGI, parental education, caregiving practices, and access to nutrition information are among the major determinants of stunting.^11^ Despite nationwide programmes promoting health and cross-sectoral efforts to reduce stunting, knowledge and attitude gaps among mothers continue to pose barriers.^29,30^ Recent research also affirms that culture, family planning, behaviour, and sanitation knowledge remain central drivers of stunting reduction.^31^

This research features several limitations. Its cross-sectional design renders it impossible to perform causal inferences, and the focus on Musi Rawas limits generalisability to urban environments or regions with different socioeconomic and cultural conditions. Additionally, certain variables, including maternal nutritional status during pregnancy, access to healthcare and cultural feeding practices, were not measured and should be included in future studies to provide a more comprehensive understanding of stunting determinants in Indonesia.

## Conclusion

This study demonstrates that the incidence of stunting is high in this rural community in South Sumatra. Low maternal knowledge levels, low maternal educational levels, and negative maternal attitudes are major predictors of poor stunting prevention methods. These findings highlight the necessity of primary healthcare intervention models that prioritise maternal health literacy and promote supportive caregiving practices. Strengthening nutrition and childcare counselling in routine Posyandu (integrated health post) activities, incorporating stunting prevention messages into school-based health promotion, and training frontline health workers are necessary strategies to improve maternal practices. Effective implementation requires coordinated participation from the District Health Office and Puskesmas (community health centres), village authorities, schools, and Posyandu volunteers. Enhancing community-based and primary care platforms can contribute to reducing stunting and enhancing child health outcomes in rural South Sumatra.

## Data sharing statement

All data generated and analysed in the study are available upon reasonable request.

**How does this paper make a difference in general practice?**

- The study identifies specific maternal factors (knowledge, education and, attitudes) affecting stunting in rural Indonesia, providing evidence for region-specific health interventions.
- The research highlights the impact of maternal knowledge and attitudes, emphasising the critical role of maternal empowerment and education in stunting prevention strategies.
- The findings offer actionable data to guide health practitioners in targeting maternal education and awareness programmes to improve child health outcomes.
